# Caecal volvulus in an adolescent African male: A case report and brief review of the literature

**DOI:** 10.11604/pamj.2014.17.92.3946

**Published:** 2014-02-06

**Authors:** Clifford Mwita, Johnstone Muthoka, Powell Kanina, Phillip Mulingwa

**Affiliations:** 1 Department of Surgery, Thika Level 5 Hospital, Thika, Kenya; 2 Joanna Briggs Institute Affiliate Centre, Kenya

**Keywords:** Caecal volvulus, Africa, Acute abdomen

## Abstract

Caecal volvulus is an infrequent clinical condition caused by an axial twist of ascending colon, caecum and terminal ileum around the mesenteric pedicle. This article presents the case of a 16-year old African male from Kenya who presented to the emergency department with acute onset right sided lower abdominal pain diagnosed intra-operatively as caecal volvulus. The rare nature of the condition, the need for a high index of suspicion and surgical management are highlighted.

## Introduction

Caecal volvulus is caused by an axial twist of the caecum, ascending colon and terminal ileum around the mesenteric pedicle [1]. It is an infrequently encountered condition with an annual incidence of 2.8 - 7.8 cases per million people per year, accounts for 1-1.5% of all adult intestinal obstructions and up to 40% of all volvulus involving the colon [1]. Depending on the presence of colon viability and intestinal gangrene, mortality ranges from 10-40% [2].

Due to its infrequent occurrence, a definitive diagnosis of caecal volvulus is often difficult to make [3]. Abdominal CT scan, barium enema and colonoscopy have been shown to be superior to plain abdominal radiographs in establishing a diagnosis but they are still not specific enough for making a definitive diagnosis [1]. Surgery is often necessary for both definitive diagnosis and management.

## Patient and observation

A 16-year old African male presented to the casualty department of Thika Level 5 Hospital with acute onset right sided lower abdominal pain that had lasted for approximately six hours. He identified no provoking factors but reported that the pain was so severe he could not walk. There was nausea and loss of appetite but no vomiting. He had not passed stool for 24 hours prior to the onset of the abdominal pain.

On physical examination, he was visibly in pain with a blood pressure of 110/70 mmHg, pulse rate of 88 beats per minute, respiratory rate of 19 cycles per minute and temperature of 36.90C. Abdominal examination revealed a non-distended abdomen with right lower quadrant tenderness but no palpable abdominal masses. A presumptive diagnosis of acute appendicitis was made. The white blood cell count was 5.8x109/uL. The blood urea, electrolytes and creatinine levels and dipstick urinalysis were normal. An abdominal ultrasound concluded there was appendicitis despite a modified Alvarado score of 2. Given the equivocal diagnosis and worsening abdominal pain, a decision was made for an exploratory laparotomy.

**Figure 1:**
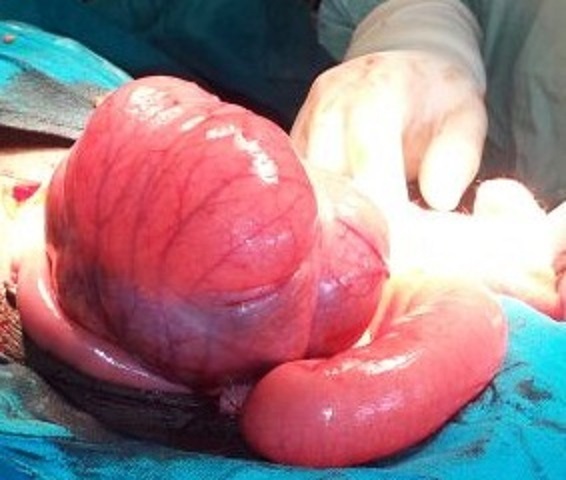
Photograph showing distended caecum with terminal ileum adjacent to it on the right side

**Figure 2:**
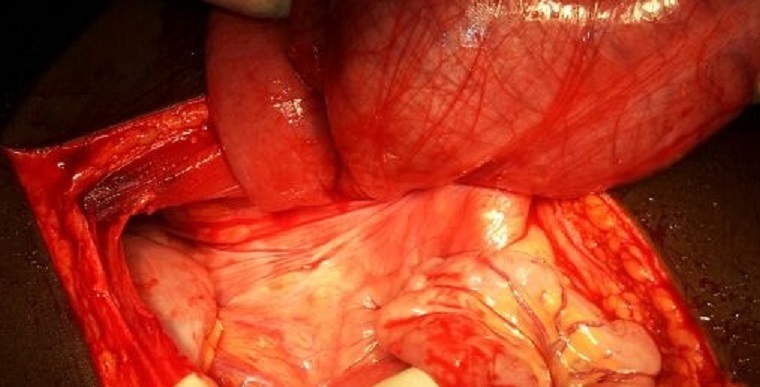
Photograph showing caecal volvulus with the point of twisting for the caecum and terminal ileum being visible

**Figure 3:**
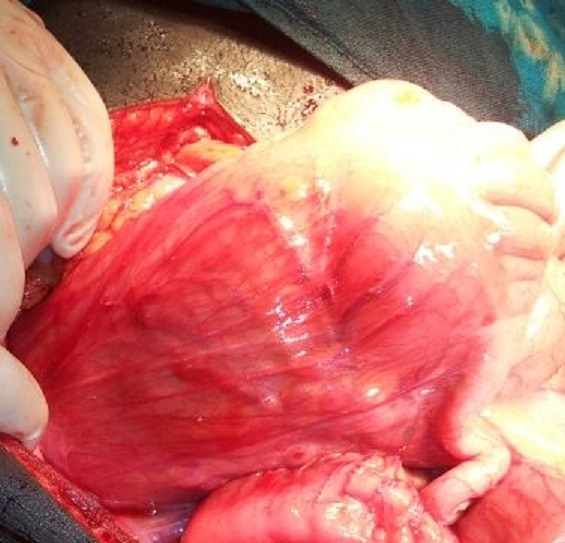
Photograph of the de-torsioned caecal volvulus showing elongated caecal mesocolon

Intraoperative findings were a distended ascending colon, cecum and terminal ileum all twisted around the mesenterybut with no gut gangrene (Figure 1, Figure 2). After detorsion, increased caecal mobility was noted as evidenced by an elongated right mesocolon (Figure 3). A right hemicolectomy with ileo-transverse anastomosis was done. The patient recovered uneventfully and was discharged on the fifth post-operative day with follow up scheduled at the surgical outpatient clinic.

## Discussion

Caecal volvulus is thought to result from increased caecal mobility in people with inadequate right colon fixation during embryogenesis [1, 4]. This leads to an elongated mesocolon with attendant increase in potential for volvulus formation. Between 11% and 25% of the population may have an elongated mesocolon [1]. However, the large difference between incidence of mobile caecum and caecal volvulus suggests that factors other than anatomical susceptibility are at play [1] and additional conditions such as history of prior abdominal surgery [5], high fiber intake [3], adynamic ileus and chronic constipation [6] are known to predispose to the condition.

Acute caecal volvulus typically presents with a clinical picture indistinguishable from acute, uncomplicated small bowel obstruction [5, 7]. However, atypical presentations have been reported. Browne [8] reports the case of an adolescent female with right lower quadrant abdominal pain that resulted in multiple inconclusive emergency department visits before a diagnosis of caecal volvulus was reached. Our patient's presentation was also atypical since he presented acutely and with no features suggestive of obstruction. Acute caecal volvulus may also present in elderly patients already hospitalized for other illnesses and a heightened clinical suspicion is warranted [9].

Besides the acute presentation, caecal volvulus may also have a recurrent intermittent illness termed mobile cecum syndrome (MCS) [10] which presents with chronic intermittent abdominal pain with spontaneous resolution after passage of flatus [1]. There may be mild right sided abdominal tenderness. MCS is an identifiable predecessor in 50% of patients with acute caecal volvulus [10]. We elicited no features of MCS in our patient on further history taking.

Laboratory values are often unremarkable in patients with caecal volvulus [1], as was the case with our patient. However, with advancedobstruction, they are useful markers for electrolyte disturbances and inflammatory or infectious changes [1]. Radiographic abnormalities are present in nearly all patients with acute caecal volvulus and include caecal dilatation, air fluid level and small bowel dilatation [1]. However, these features are non-specific and should only help to raise suspicion of caecal volvulus leading to subsequent confirmation by barium enema, computed tomography (CT) scan, colonoscopy or surgery [1, 4, 7].

Barium enema is 88% accurate for volvulus [7] and enables visualization of the distal colon to exclude contributory abnormalities [1]. It has occasional success in reduction of volvulus [5]. However, it is time consuming and has potential for contrast extravasation and thus is unsuitable for critically ill patients [4, 7, 9]. CT scan is replacing barium enema as the imaging modality of choice in the diagnosis of acute caecal volvulus. The “coffee bean”, “bird beak” and “whirl” signs are pathoneumonic for caecal volvulus when visualized on CT scan [11]. Visualization of a gas filled appendix has also been noted as a CT scan finding associated with caecal volvulus [11] and this may explain the erroneous ultrasound diagnosis of appendicitis in our patient. Unlike the case in sigmoid volvulus, the success rate for colonoscopic reduction of caecal volvulus is only 30% [2] and given the potential for colonic perforation and delays in operative treatment, colonoscopy is generally not recommended in the management of caecal volvulus [4, 9].

The mainstay of therapy for acute caecal volvulus is surgery for the correction of intestinal obstruction [1, 3]. We performed a right hemicolectomy with ileo-transverse anastomosis despite the absence of gut strangulation. As has been previously suggested [1], this procedure eliminates the possibility of recurrence. Other treatment options include operative detorsion, caecopexy and caecostomy tube placement [1, 4]. However, the high recurrence rate of volvulus after these procedures rules out their use despite lower morbidity and mortality rates. Nonetheless, the advent of advanced peri-operative care and laparoscopic techniques has led to colectomy being more popular [1, 4].

## Conclusion

Although caecal volvulus is an infrequent clinical condition, it should be borne in mind when evaluating patients with acute abdomen, regardless of age. In the absence of a conclusive preoperative diagnosis surgery is both diagnostic and therapeutic.

## References

[1] Consorti ET, Liu TH (2005). Diagnosis and treatment of caecal volvulus. Postgrad Med J..

[2] Renzulli P, Maurer CA, Netzer P, Buchler MW (2002). Preoperative colonoscopic derotation is beneficial in acute colonic volvulus. Dig Surg..

[3] Pulvirenti E, Palmieri L, Toro A, Di Carlo I (2010). Is laparotomy the unavoidable step to diagnose caecal volvulus?. Ann R Coll Surg Engl..

[4] Madiba TE, Thomson SR (2002). The management of cecal volvulus. Dis Colon Rectum..

[5] O'Mara CS, Wilson THJSGL, Stonesifer GL, Cameron JL (1979). Cecal volvulus: analysis of 50 patients with long-term follow-up. Ann Surg..

[6] Radin DR, Halls JM (1986). Cecal volvulus: a complication of colonoscopy. Gastrointest Radiol..

[7] Rabinovici R, Simansky DA, Kaplan O, Mavor E, Manny J (1990). Cecal volvulus. Dis Colon Rectum..

[8] Browne N (2010). Cecal volvulus in adolescence presenting as recurring visits for abdominal pain. West J Emerg Med..

[9] Friedman JD, Odland MD, Bubrick MP (1989). Experience with colonic volvulus. Dis Colon Rectum..

[10] Donhauser JL, Atwell S (1949). Volvulus of the cecum with a review of 100 cases in the literature and a report of six new cases. Arch Surg..

[11] Moore CJ, Corl FM, Fishman EK (2001). CT of cecal volvulus: unraveling the image. AJR Am J Roentgenol..

